# Human Papillomavirus Vaccination Among Young Adults Before and During the COVID-19 Pandemic

**DOI:** 10.1001/jamanetworkopen.2023.56875

**Published:** 2024-02-20

**Authors:** Kalyani Sonawane, Ashvita Garg, Eric G. Meissner, Haluk Damgacioglu, Elizabeth Hill, Alan G. Nyitray, Ashish A. Deshmukh

**Affiliations:** 1 Department of Public Health Sciences, College of Medicine, Medical University of South Carolina, Charleston; 2 MUSC Hollings Cancer Center, Charleston, South Carolina; 3 Department of Psychiatry and Behavioral Medicine, Medical College of Wisconsin, Milwaukee; 4 Department of Medicine, College of Medicine, Medical University of South Carolina, Charleston

## Abstract

This cross-sectional survey study assesses the self-reported human papillomavirus vaccination rate by sociodemographic characteristics in adults aged 18 to 26 years from 2018 to 2022.

## Introduction

In the US, the human papillomavirus (HPV) vaccine is routinely recommended as catch-up vaccination for adults aged 18 to 26 years who were not adequately vaccinated at a younger age.^1^ Before the COVID-19 pandemic started in March 2020, a steady increase in HPV vaccination coverage in this population was reported.^2^ However, coverage during the pandemic remains unknown. We compared national HPV vaccination coverage among young adults before and during the pandemic and assessed recent vaccination rates among racial, ethnic, and sexual minority groups and other sociodemographic subgroups.

## Methods

We analyzed data for participants aged 18 to 26 years in the 2018, 2019, and 2022 National Health Interview Survey (NHIS), a nationally representative survey of a civilian noninstitutionalized population.^3^ Trained interviewers administered the survey after obtaining participants’ informed consent. Sociodemographic information and HPV vaccination status were self-reported; vaccination status was not collected in 2020 and 2021. The Medical University of South Carolina Institutional Review Board deemed this cross-sectional survey study exempt from review and informed consent because it used deidentified publicly available data. We followed the AAPOR reporting guideline.^4^

We defined coverage as receipt of 1 or more doses of HPV vaccine before age 27 years. Proportions and representative population counts were estimated using NHIS sampling weights. Multivariable models estimated prevalence ratios. Statistical significance was tested at *P* < .05. All analyses were performed with SAS 9.4, specifically SURVEY procedures to incorporate sampling weights and adjust for the complex survey design.

## Results

A total of 2159 (estimated 33.6 million) adults aged 18 to 26 years (50.5% females [16.9 million], 49.5% males [16.6 million]; 23.0% Hispanic, 12.9% non-Hispanic Black, 53.4% non-Hispanic White, 10.7% other race and ethnicity [non-Hispanic American Indian or Alaska Native, non-Hispanic Asian, any other groups, and other single and multiple races]) with HPV vaccination information were identified in 2022. Overall, 47.4% reported receiving 1 or more vaccine doses. Unlike the increase in HPV vaccination rate from 2018 to 2019 (39.9% to 47.0%; *P* < .001), no significant change was observed from 2019 to 2022 (Figure). In 2022, more females than males were vaccinated (57.2% vs 37.3%; *P* < .001).

**Figure.**
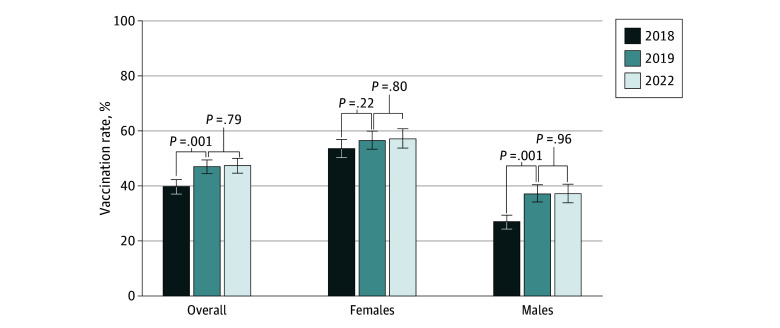
Human Papillomavirus (HPV) Vaccination Coverage Among US Adults Aged 18 to 26 Years for 2018, 2019, and 2022 Error bars represent 95% CIs. *P* values were calculated using survey design–adjusted Wald *F* test. Participants who responded yes to the question “Have you ever received the HPV vaccine?” were identified as vaccinated with 1 or more HPV vaccine doses. Sex was not reported for 1 participant in 2022.

In 2022, coverage among Hispanic, non-Hispanic Black, and participants of other races and ethnicities was similar compared with non-Hispanic White individuals (Table). Coverage was higher among lesbian, gay, bisexual, and other sexual orientation (LGB+) group than heterosexual females (70.6% vs 53.6%; *P* < .001) and higher among gay, bisexual, and other sexual orientation (GB+) group than heterosexual males (52.7% vs 36.2%; *P* = .02). Coverage was lower among uninsured males and females (20.0% and 33.9%; *P* < .001) vs their insured counterparts (40.9% and 60.6%; *P* < .001). Coverage was higher among females and males with associate or bachelor’s degrees (68.2% and 45.4%; *P* < .001) vs those with high school or lower educational level (43.7% and 29.5%; *P* < .001). No significant differences were detected by region or urbanicity. Findings were consistent in multivariable analyses.

**Table.** HPV Vaccination Coverage in 2022 Among Adults Aged 18 to 26 Years by Sociodemographic Characteristics^a^

Characteristic^b^	Female HPV vaccination coverage		Male HPV vaccination coverage	
	% (95% CI)	Prevalence ratio (95% CI)	% (95% CI)	Prevalence ratio (95% CI)
Sexual orientation^c^				
Heterosexual	53.6 (49.7-57.6)	1 [Reference]	36.2 (32.6-39.9)	1 [Reference]
LGB+	70.6 (64.0-77.1)	1.33 (1.17-1.42)	52.7 (39.9-65.5)	1.44 (1.01-1.77)
Race and ethnicity^d^				
Hispanic	50.2 (44.1-56.4)	0.91 (0.71-1.07)	34.3 (28.1-40.5)	0.96 (0.69-1.22)
Non-Hispanic Black	52.9 (44.0-61.8)	0.88 (0.63-1.08)	30.0 (20.5-39.6)	0.81 (0.49-1.16)
Non-Hispanic White	59.7 (54.9-64.4)	1 [Reference]	40.2 (35.4-45.1)	1 [Reference]
Other^e^	66.0 (56.3-75.6)	1.13 (0.88-1.28)	37.1 (27.4-46.8)	0.92 (0.60-1.24)
Insurance status				
Insured	60.6 (57.1-64.0)	1 [Reference]	40.9 (37.1-44.8)	1 [Reference]
Not insured	33.9 (24.5-43.4)	0.65 (0.42-0.89)	20.0 (12.3-27.6)	0.50 (0.28-0.81)
Educational level				
≤High school diploma	43.7 (38.0-49.4)	1 [Reference]	29.5 (24.9-34.0)	1 [Reference]
Some college	65.6 (59.5-71.6)	1.47 (1.26-1.58)	46.7 (39.4-54.0)	1.42 (1.08-1.71)
Associate or bachelor’s degree	68.2 (63.1-73.3)	1.54 (1.36-1.62)	45.4 (39.0-51.8)	1.35 (1.02-1.63)
≥Master’s degree	58.6 (41.1-76.1)	1.29 (0.79-1.61)	53.3 (30.0-76.5)	1.75 (0.87-2.35)
Region				
Northeast	60.3 (51.8-68.8)	1 [Reference]	39.2 (31.2-47.2)	1 [Reference]
Midwest	60.9 (54.1-67.7)	1.00 (0.73-1.16)	38.5 (31.0-46.1)	1.02 (0.66-1.31)
South	54.4 (49.0-59.8)	1.00 (0.73-1.15)	36.1 (30.1-42.1)	1.05 (0.71-1.32)
West	56.4 (49.6-63.3)	0.90 (0.62-1.10)	36.5 (29.6-43.3)	1.01 (0.66-1.31)
Area of residence				
Metropolitan	58.1 (54.6-61.7)	1 [Reference]	38.6 (34.8-42.3)	1 [Reference]
Nonmetropolitan	50.2 (41.0-59.4)	0.84 (0.61-1.05)	28.4 (18.6-38.3)	0.75 (0.44-1.13)

Abbreviations: HPV, human papillomavirus; LGB+, lesbian, gay, bisexual, and other sexual orientation.

^a^ The HPV vaccination status was ascertained using this question in the National Health Interview Survey (NHIS): “Have you ever received the HPV vaccine?” Participants who responded yes were identified as vaccinated with 1 or more HPV vaccine doses.

^b^ Information on race and ethnicity (n = 1), sexual orientation (n = 175), insurance status (n = 25), educational level (n = 11), region (n = 1), and area of residence (n = 1) was missing for some participants.

^c^ Participants were combined into a single LGB+ category due to small sample sizes. Nonbinary and transgender individuals could not be identified in the NHIS dataset.

^d^ Race and ethnicity were collected separately. A combined recoded variable on race and ethnicity was provided by the NHIS and included imputed data when race and Hispanic origin were unknown. We used the race and ethnicity variable provided in the NHIS dataset.

^e^ The other category included non-Hispanic American Indian or Alaska Native, non-Hispanic Asian, any other groups, and other single and multiple races.

## Discussion

The results of this study suggest that human papillomavirus vaccination coverage among young adults did not increase during the COVID-19 pandemic compared with prior years.^2^ This finding likely reflects pandemic-related disruptions in initiating the HPV vaccine among young adults. We observed higher vaccination rates among LGB+ and GB+ groups. Given that sexual minority groups have higher HPV infection prevalence, are at an elevated risk for HPV-related cancers, and are unlikely to benefit from herd protection,^5,6^ higher vaccination rates are reassuring. Low rates among uninsured participants underscore the importance of making the HPV vaccine accessible to uninsured individuals.

Study limitations include the self-reported nature of NHIS data; recall bias; and lack of data on transgender and nonbinary individuals, HIV or immunocompromised status, and total number of doses received, which prohibited us from presenting up-to-date status. Nevertheless, findings from the nationally representative dataset highlight a hiatus in the progress toward coverage goals among young adults. Moreover, disaggregation of vaccination rates by sociodemographics helped discern subgroups that could benefit from targeted outreach.

## Notes

Supplement.
Data Sharing Statement
