# Supplementary material for: Human Papillomavirus Vaccination Among Young Adults Before and During the COVID-19 Pandemic
Source: JAMA Netw Open. 2024 Feb 20;7(2):e2356875. doi: 10.1001/jamanetworkopen.2023.56875 (PMC10879942; doi:10.1001/jamanetworkopen.2023.56875)
Supplement: Supplement. — Data Sharing Statement [file jamanetwopen-e2356875-s001.pdf]

Data Sharing Statement

Sonawane. Human Papillomavirus Vaccination Among Young Adults Before and During the COVID-19 Pandemic. *JAMA Netw Open*. Published February 20, 2024. doi:10.1001/jamanetworkopen.2023.56875

Data
Data available: Yes
Data types: Deidentified participant data, Data dictionary
How to access data: https://www.cdc.gov/nchs/nhis/index.htm
When available: With publication

Supporting Documents
Document types: None

Additional Information
Who can access the data: Anyone
Types of analyses: Any purpose
Mechanisms of data availability: https://www.cdc.gov/nchs/nhis/index.htm
